# A Computational Analysis of Stoichiometric Constraints and Trade-Offs in Cyanobacterial Biofuel Production

**DOI:** 10.3389/fbioe.2015.00047

**Published:** 2015-04-20

**Authors:** Henning Knoop, Ralf Steuer

**Affiliations:** ^1^Institut für Theoretische Biologie, Humboldt-Universität zu Berlin, Berlin, Germany

**Keywords:** flux-balance analysis, metabolic modeling, cyanobacteria, *Synechocystis* sp. PCC 6803, microbial cell factories, photosynthesis

## Abstract

Cyanobacteria are a promising biological chassis for the synthesis of renewable fuels and chemical bulk commodities. Significant efforts have been devoted to improve the yields of cyanobacterial products. However, while the introduction and heterologous expression of product-forming pathways is often feasible, the interactions and incompatibilities of product synthesis with the host metabolism are still insufficiently understood. In this work, we investigate the stoichiometric properties and trade-offs that underlie cyanobacterial product formation using a computational reconstruction of cyanobacterial metabolism. First, we evaluate the synthesis requirements of a selection of cyanobacterial products of potential biotechnological interest. Second, the large-scale metabolic reconstruction allows us to perform *in silico* experiments that mimic and predict the metabolic changes that must occur in the transition from a growth-only phenotype to a production-only phenotype. Applied to the synthesis of ethanol, ethylene, and propane, these *in silico* transition experiments point to bottlenecks and potential modification targets in cyanobacterial metabolism. Our analysis reveals incompatibilities between biotechnological product synthesis and native host metabolism, such as shifts in ATP/NADPH demand and the requirement to reintegrate metabolic by-products. Similar strategies can be employed for a large class of cyanobacterial products to identify potential stoichiometric bottlenecks.

## 1 Introduction

Cyanobacteria are a promising resource for the renewable synthesis of various chemical compounds. High efforts are currently devoted to expand the range of potential products suitable for production using modified cyanobacteria as a biological chassis. Starting with the synthesis of ethanol in cyanobacteria by Deng and Coleman (1999) and its later improvements (Dienst et al., 2014), the repertoire of synthetic cyanobacterial biosynthesis is continuously increasing and currently encompasses of a broad range of compounds, including isoprene (Lindberg et al., 2010), ethylene (Takahama et al., 2003; Guerrero et al., 2012; Ungerer et al., 2012; Jindou et al., 2014), lactate (Angermayr et al., 2012; Joseph et al., 2013; Varman et al., 2013b), 1-butanol (Lan and Liao, 2011, 2012), 2,3-butanediol (Oliver et al., 2013; Savakis et al., 2013), isobutyraldehyde (Atsumi et al., 2009), isobutanol (Atsumi et al., 2009; Varman et al., 2013a), 3-hydroxybutryrate (Wang et al., 2013), mannitol (Jacobsen and Frigaard, 2014), squalene (Englund et al., 2014), as well as several high value products (Ducat et al., 2011).

In principle, the metabolic modification of cyanobacteria, in particular, of the laboratory strain *Synechocystis* sp. PCC 6803, for the production of a desired compound is not difficult. However, such an initial modification typically results in low product yields. Strategies to improve yield, genetic stability, and robustness of production strains are therefore urgently needed. As yet, the development of such strategies is often based on trial and error, and is guided only by the experience of researchers. Only few systematic frameworks exist that allow to identify and rank suitable modifications to improve desired properties of a production strain.

A possible strategy overcome some of the current bottlenecks in strain design is the use of computational *in silico* models to rapidly test possible modifications of potential production strains. In particular, the use of large-scale models of microbial metabolism has become a common tool in heterotrophic biotechnology, see Zomorrodi et al. (2012) for an overview, with as yet only few applications in cyanobacterial biotechnology (Montagud et al., 2011; Nogales et al., 2013; Sengupta et al., 2013; Vu et al., 2013; Erdrich et al., 2014). However, an increasing number of large-scale reconstructions of cyanobacterial strains are now available (Knoop et al., 2013; 2010; Montagud et al., 2010; Yoshikawa et al., 2011; Nogales et al., 2012; Saha et al., 2012; Vu et al., 2012), setting the stage for further applications of predictive strain design. Such large-scale metabolic reconstructions seek to provide a comprehensive compendium of all biochemical interconversions of small molecules (metabolites) taking place within a cell, and therefore provide a highly useful knowledge base for a systematic computational interrogation of cyanobacterial metabolism. Starting point of a metabolic reconstruction is usually the annotated genome sequence. Based on the initial set of annotated enzymes, the completeness of synthesis routes for all known cellular constituents can be tested and, if necessary, additional reactions can be identified and added to the reconstruction (Steuer et al., 2012; Knoop et al., 2013). Current reconstructions of cyanobacterial metabolism are based on extensive manual curation and can be expected to provide a reasonably comprehensive picture of cyanobacterial metabolism. Once established, a metabolic reconstruction can serve a multitude of purposes, from integrating large-scale data to probing potential production strains using *in silico* knockouts. A large number of computational tools are now available that allow for efficient and fast interrogation of reconstructed metabolic networks (Zomorrodi et al., 2012).

In this work, we utilize a recent reconstruction of the cyanobacterium *Synechocystis* sp. PCC 6803 to investigate key properties of cyanobacterial product synthesis. In particular, the large-scale reconstruction offers the unique possibility to derive features of production pathways in the context of the entire cellular metabolism. Augmenting previous work (Kämäräinen et al., 2012; Erdrich et al., 2014), we are interested in the maximal production yields of various potential fuels and bulk products, in the trade-offs between cellular growth and product synthesis, as well as in the identification of reactions that may hinder or limit the synthesis of a desired product. In contrast to explicit kinetic models of cellular pathways, our analysis is based only on knowledge of network stoichiometry – information that is readily and reliably available for an increasing number of cyanobacterial strains. We are specifically interested in changes in reaction fluxes that must occur to enable a transition from a growth-only wildtype (WT) phenotype to a production-only phenotype. The metabolic model allows us to perform such hypothetical *in silico* transitions within the metabolic flux space, and thereby allows us to identify potential bottlenecks and incompatibilities between product synthesis and native metabolism. In particular, the computational analysis allows us to consider hypothetical production scenarios with high product flux. We show that high product flux inevitable induces metabolic by-products that must be reintegrated into native metabolism, necessitating changes in reaction fluxes that are not part of the synthesis pathway itself. We argue that such potential incompatibilities only become manifest in a situation where host metabolism is dominated by product synthesis. In concurrence with earlier studies (Erdrich et al., 2014), we further show that shifts in the ATP/NADPH utilization ratio are promising targets for biotechnological modifications.

## 2 Results

### 2.1 Stoichiometric properties of cyanobacterial biofuels

**Figure 1:**
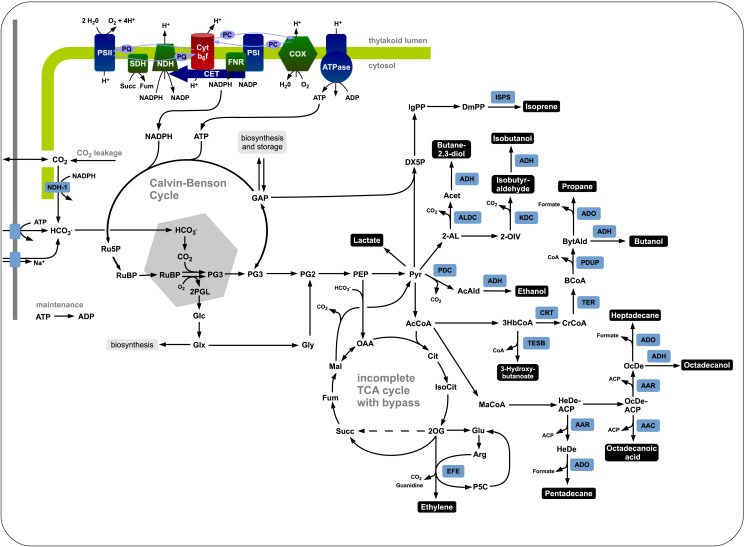
**The central carbon metabolism of *Synechocystis* sp. PCC 6803 and associated production pathways**. Inorganic carbon is assimilated by Rubisco in the carboxysome using energy and redox equivalent harvested by the photosynthetic light reactions. The product 3-phosphoglycerate serves as precursor for product synthesis. Shown are key pathways toward ethanol, lactate, isoprene, ethylene, butane-2,3-diol, isobutanol, isobutyraldehyde, butanol, propane, 3-hydroxybutyrate, heptadecane, octadecanol, octadecanoic acid, and pentadecane.

We evaluate the synthesis of 12 potential products of biotechnological interest whose production using cyanobacteria has been demonstrated, or at least is biologically feasible. Figure 1 gives an overview on the respective production pathways in the context of cyanobacterial central metabolism. Specifically, we evaluate the stoichiometric properties of the synthesis of ethanol, ethylene, lactate, propane, butanol, isoprene, butane-2,3-diol, isobutyraldehyde, isobutanol, pentadecane, heptadecane, and octadecanol. The respective synthesis pathways are summarized in the Materials and Methods and are introduced into an extended metabolic reconstruction of the cyanobacterium *Synechocystis* sp. PCC 6803 (Knoop et al., 2013). First, we are interested in basic stoichiometric properties of the respective synthesis pathways. The suitability of a potential fuel is determined by several factors. From a biological perspective, an important criterion is the maximal stoichiometric yield of the respective product, relative to a given light input and relative to the specific lower heating value (LHV). Extending previous work (Kämäräinen et al., 2012), Table 1 shows the maximal stoichiometric yield of each fuel, as well as several further characteristic stoichiometric properties. We note that the maximal energetic yields of the different products, relative to the respective average heating value, are similar. In particular, products associated with longer pathways do not necessarily have significantly lower energetic yield. We note, however, that Table 1 indicates maximal yield only, as obtained computationally by assuming lossless stoichiometric interconversions irrespective of pathway length.

**Table 1:** **Stoichiometric properties of cyanobacterial biofuel synthesis**.

	# React	Photons	NADPH	ATP	ATP/NADPH	O_2_	RuBisco	CO_2_	Flux	Yield
									[mmol gDW^−1^h^−1^]	[J gDW^−1^h^−1^]
Ethanol	56	24	6	7	1.17	3	3	2	0.649	0.889
Ethylene	83	61	9.5	19.5	2.05	6	6	2.5	0.255	0.360
Lactate	55	24	6	7	1.17	3	3	3	0.649	0.884
Propane	63	50	13	12	0.92	5	4	3	0.311	0.691
1-Butanol	62	48	12	12	1	6	4	4	0.324	0.866
Isoprene	66	56	13	17	1.31	7	6	5	0.278	0.887
1-Octadecanol	93	216	54	62	1.15	27	18	18	0.072	0.852
Heptadecane	94	218	53	62	1.13	26	18	17	0.071	0.811
Pentadecane	90	194	49	55	1.12	23	16	15	0.080	0.807
2,3-Butanediol	60	44	11	14	1.27	5.5	6	4	0.354	0.871
Isobutyraldehyde	60	44	11	14	1.27	5.5	6	4	0.354	0.874
Isobutanol	62	48	12	14	1.17	6	6	4	0.324	0.866
Biomass	459	≈539	≈90	≈191	≈2.13	≈62.7	≈42.0	≈41.5	≈0.029	–

*Estimated values are based on a recent reconstruction of Synechocystis *sp*. PCC 6803. For each product, only the pathway with maximal yield is taken into account and requirements for cellular growth and maintenance are neglected. Shown is the number of active reactions required for the synthesis of the respective product, the minimal number of required photons per molecule of product, O_2_ release, ATP, and NADPH requirements, flux through the Rubisco reaction, as well as the maximal synthesis flux using a light input of 15.57 μmol photons gDW^−1^h^−1^ (corresponding to a WT doubling time of approximately 24 h), as well as the resulting energy yield based on lower heating value (LHV). As expected, for a given light intensity, there is a clear relationship between maximal synthesis rate, demand of photons per molecule of product, number of active reactions, and size of the product in terms of carbon molecule. We note that in the case of ethylene the by-product guanidine is not further metabolized. See Section “Materials and Methods” for an alternative estimation of reported values taking complete reintegration of guanidine into account*.

A known exception to the approximately similar maximal energetic yields is the synthesis of the alkene ethylene. We consider synthesis of ethylene via the ethylene-evolving enzyme (EFE), using 2-oxoglutarate and arginine as substrates and producing succinate, P5C, guanidine, and CO_2_ as by-products. In particular, the release of 7 CO_2_ makes the enzyme highly inefficient for phototrophic fuel production.

Likewise, the alkane propane has a slightly lower maximal stoichiometric yield because of the release of formate by the aldehyde decarbonylase. The maximal energetic yield of propane is significantly below that of 1-butanol, even though both synthesis pathways are almost identical. Otherwise, notwithstanding the overall similarity of maximal energetic yield, small differences in maximal yield can be attributed to pathway length, as well as NADPH and ATP utilization. Ethanol has the highest maximal energetic yield, whereas the value tends to be slightly lower for larger molecules.

Additional stoichiometric properties summarized in Table 1 are the number of active reactions during fuel synthesis, the number of photons consumed per molecule product, the demand for ATP and NADPH, the release of oxygen, as well as the amount of carbon fixed by Rubisco. The latter differs from the amount of carbon molecules per molecule of fuel due to, as in the case of ethylene, formation of by-products and release of CO_2_ in production pathways. A property of importance is the ATP/NADPH ratio required for the synthesis of a desired product, ranging from approximately 0.92 for propane up to more than 2.0 for ethylene. In contrast, the ATP/NADPH ratio of the photosynthetic linear electron chain (ETC) is approximately 1.29, the computationally estimated ratio for the synthesis of biomass is 2.13. Notwithstanding significant caveats in its estimation (see Materials and Methods), the difference between the required ATP/NADPH demand reflects the intuitive notion that the synthesis of biomass, for example, the translation of amino acids into proteins, requires far more ATP per mole assimilated inorganic carbon than the excretion of the respective precursor molecules as a bioproduct. Indeed, our recent study on cyanobacterial strain design (Erdrich et al., 2014) concluded that the computationally identified knockout or overexpression strategies mostly seek to modify the ATP/NADPH ratio as a suitable strategy to enhance metabolic flux toward a given product. The rationale behind these modifications is to enforce the synthesis of desired products as electron sinks – analogously to heterotrophic fermentation pathways. Along these lines, the study of Erdrich et al. (2014) suggests that even ATP-wasting by heterologously expressed futile cycles may be a suitable strategy to increase the relative amount of NADPH, and hence force metabolism toward the synthesis of ethanol or other fuels with low ATP/NADPH ratio – a theme that will recur below.

As noted above, Table 1 represents the maximal stoichiometric yield using native metabolism, augmented by the heterologously production pathway, and assuming lossless interconversions. It must be expected that actual experimental yields are significantly below these values. Likewise, we note that the computational evaluation considers synthesis via the pathway with highest stoichiometric yield. Alternative pathways with lower yield, as well as catalytic efficiencies of enzymes, product inhibition, and potential toxicity of by-products are not considered within the computational model.

### 2.2 Trade-offs between growth and product synthesis

**Figure 2:**
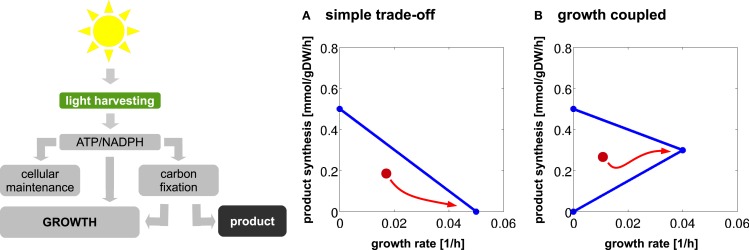
**The trade-offs between growth and product synthesis**. Light energy harvested by the photosynthetic light reaction is either used for cellular growth, for growth-independent maintenance, or diverted into product synthesis. **(A)** A simple trade-off between growth and product synthesis. **(B)** Growth-coupled product synthesis. In the latter case, product synthesis is an inevitable by-product of cellular growth. The red arrow denotes a possible evolutionary trajectory maximizing growth rate in a continuously growing culture following an initial metabolic modification. For a simple trade-off, the organism will eventually revert back to a growth-only phenotype.

**Figure 3:**
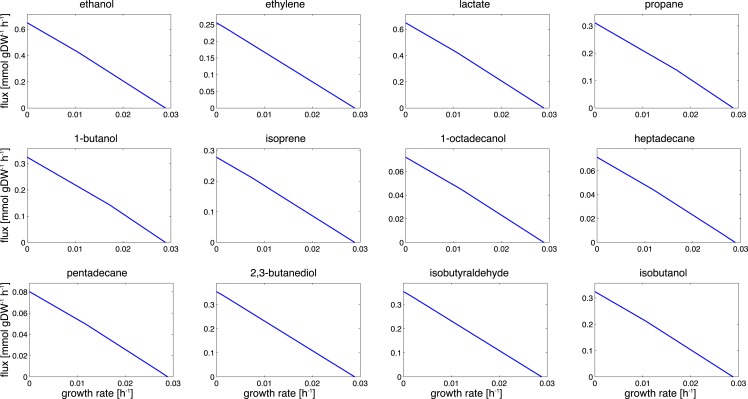
**Trade-offs between growth and product formation**. Shown is the maximal product flux for a given growth rate under constant light. Starting with a growth-only phenotype, the required growth rate is gradually reduced and resources are diverted toward the synthesis of the product. For a given light intensity, all flux rates that are bound by the trade-off curves are energetically and stoichiometrically feasible, the curves itself represent the line of maximal resource utilization. The observed shapes are slightly convex. We note that the computational analysis does not incorporate the sink effect, where the introduction of an additional carbon sink may also increase the total photosynthetic rate.

Product synthesis typically takes place in the context of a growing cell. Energy harvested in the photosynthetic light reactions can either be used for cellular growth, for growth-independent cellular maintenance, or for the production of a desired compound. Assuming an approximately constant requirement for growth-independent maintenance and a constant light availability, we therefore expect a trade-off between cellular growth and product synthesis, as illustrated in Figure 2. Figure 3 shows the corresponding results for the 12 cyanobacterial products considered in this study. In each case, we observe a simple trade-off, albeit with slight deviations from a straight line. The joint synthesis of fuel and growth is slightly more efficient per absorbed photon, as compared to synthesis of a product alone. This difference is due to small synergies between synthesis of product and biomass. For example, by-products that are synthesized during the formation of a desired product must be either excreted or recycled within cellular metabolism. Since most by-products already have non-zero metabolic turnover during growth, the latter task is relatively straightforward to achieve in the context of a growing cell. With biofuel synthesis as the dominant flux; however, the reintegration of by-products is getting increasingly difficult. A large product synthesis flux also implies a high amount of by-products, whose further utilization requires upregulation of specific reactions compared to wildtype growth.

A further cause contributing to the small synergy between fuel synthesis and growth resides in the incompatibility of linear electron flow with the ATP/NADPH ratios required for product synthesis. Most products have an ATP/NADPH requirement below that of biomass synthesis, hence the requirements of NADPH induce an excess availability of ATP. While product synthesis is small this excess ATP is readily used for cellular growth and maintenance. As soon as product synthesis becomes the dominant metabolic process, however, excess ATP cannot be straightforwardly channeled into growth or other cellular processes and therefore diminishes overall yield.

We note that the overall shapes of the trade-off curves shown in Figure 3 are markedly different from characteristic trade-offs for heterotrophic product formation. In the latter case, there is often a strong synergy between product formation and growth, and, in some cases, even growth-coupled product synthesis. See Figure 2B for an example. The latter implies that product formation is a necessary by-product during growth. A well-known example for growth-coupled synthesis is the formation of ethanol during anaerobic heterotrophic growth. Here, ethanol serves as a sink for NADH, and energy generation without the concomitant production of ethanol is not feasible. From a biotechnological perspective, growth-coupled product formation is highly attractive as it solves the problem of genetic stability, and allows to select and optimize organisms for growth – a comparatively easier task than selecting for product formation. Therefore, a number of computational strategies have been developed that aim to engineer growth-coupled synthesis of desired products into various host organisms (Burgard et al., 2003; Zomorrodi et al., 2012). It was recently concluded (Erdrich et al., 2014) that growth-coupled synthesis is indeed also possible for cyanobacteria. The number of required metabolic modifications, however, is larger than for typical heterotrophic bacteria.

### 2.3 Metabolic transitions for biofuel synthesis

The synthesis of desired products is expected to induce changes in the host metabolism with respect to reaction fluxes and co-factor usage. We are interested, which reactions must be expected to carry different flux when comparing a growth-only phenotype and a production phenotype. To this end, we perform *in silico* experiments, analogously to the analysis of production trade-offs shown in Figure 3. Starting with a growth-only phenotype that maximizes synthesis of biomass and given a constant light availability, we gradually reduce the minimal requirement of biomass synthesis and introduce the synthesis of a desired product as a new computational objective. Correspondingly, the resulting predicted flux distribution diverts increasing resources toward the synthesis of the desired product, until growth ceases and only the product is synthesized. Throughout the transition, all changes in reaction fluxes are recorded. As demonstrated below, the evaluation of these differentially utilized reactions provides useful insight for possible modifications and potential bottlenecks associated to product synthesis.

**Figure 4:**
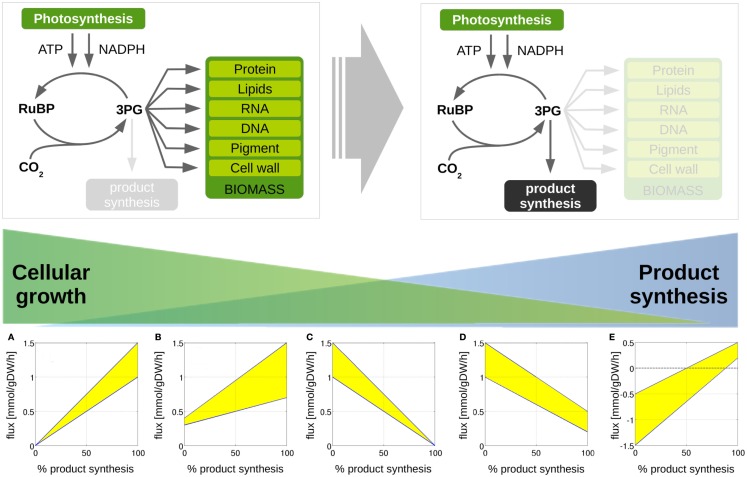
**Metabolic transitions from cellular growth to product formation**. We are interested in computationally predicted flux changes during the transition from growth-only phenotype to a production-only phenotype. We distinguish 5 different cases. **(A)** A given reaction flux is only active for product synthesis and attains a zero value for WT growth; **(B)** a reaction flux attains a non-zero value for growth, but increases for product synthesis; **(C)** a reaction flux is only active during growth and attains a zero value for product synthesis, **(D)** the predicted reaction flux for growth is larger than the flux required for product synthesis, **(E)** the reaction flux changes sign during the transition for a growth phenotype to a production phenotype. We note that predicted flux solutions are not unique. The shaded areas indicate regions of computationally equivalent solutions (flux variability).

Our approach is further illustrated in Figure 4. The following scenarios can be distinguished: first, we expect that the majority of reaction fluxes decrease during the transition to product synthesis. The synthesis of any individual compound typically requires far fewer active reactions than the synthesis of biomass, whose formation involves the formation of a broad range of cellular compounds. Reactions that only participate in biomass synthesis but are not required for the synthesis of the desired product will eventually reach zero flux in the *in silico* transition experiment. Second, there may be reactions that carry lower, but still non-zero, flux in the production-only phenotype, as compared to wildtype growth. That is, flux through these reactions is required for product synthesis, but, given a fixed light intensity, this flux is significantly lower than the value required for cellular growth. These reactions are potential candidates for down-regulation, as their enzymatic capacities in the native host organism are likely to be adapted to a wildtype growth, hence exceeds the capacity required for product synthesis. Third, in addition to reactions with decreased flux, we also expect a number of reaction fluxes to increase during the transition toward synthesis of a desired product. In particular, the synthesis reactions itself, which are often heterologously expressed and therefore do not carry any flux in the WT. Of particular interest, however, are those reaction fluxes that increase their value during product synthesis, but are not itself part of the core synthesis pathway. These reactions constitute potential bottlenecks, as their enzymatic capacities may not be sufficient to support the increased flux necessary for product synthesis. As will be shown below, these reactions fluxes are typically associated with the recycling and reintegration of by-products and the adaptation of co-factor usage for product synthesis. Finally, in a few cases, fluxes may also reverse direction during the transition from a growth-only to a product-only phenotype. Table 2 provides a summary of the number of these different cases encountered in the transition experiments for the 12 bioproducts considered here. We note that the analysis of metabolic transitions toward fuel synthesis is complicated by the fact that the computationally predicted fluxes are usually not unique. Rather, several feasible and functionally equivalent flux solutions may exist. We therefore must take flux variability into account. Unless otherwise noted, all flux changes are reported in terms of median flux.

**Table 2:** **Metabolic transitions from cellular growth to product formation**.

	Sign Change	Flux increase		Flux decrease		No change
		From flux	From zero	To flux	To zero	
Ethanol	8	63	42	8	371	63
Ethylene	9	70	42	12	360	63
Lactate	8	64	40	8	371	62
Propane	8	45	47	18	370	72
1-Butanol	9	45	46	16	370	74
Isoprene	8	74	41	8	360	63
1-Octadecanol	8	83	42	13	335	74
Heptadecane	8	82	43	13	335	75
Pentadecane	8	77	43	14	339	75
Butane-2,3-diol	8	68	42	7	369	61
Isobutyraldehyde	8	70	41	7	367	61
Isobutanol	8	68	42	7	367	63

*Changes in computationally predicted flux utilization are recorded. Shown is the number of reaction fluxes that increase, either from zero value or from a non-zero value, during the transition to a production-only phenotype, as well as the number of reaction fluxes that decrease, either to zero or to a non-zero value, during the transition to a production-only phenotype. A reaction flux is defined as unchanged if the median flux, taking flux variability into account, changes by <5%. A small number of reaction fluxes change sign during the transition*.

### 2.4 Metabolic transitions for the synthesis of ethanol

**Figure 5:**
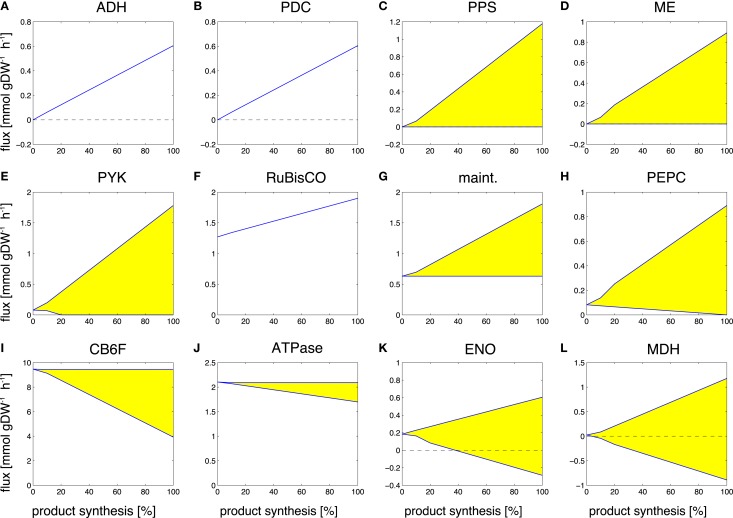
**Examples of predicted changes in reaction fluxes during the transitions from growth-only phenotype to production-only phenotype for ethanol**. Yellow areas correspond to flux variability, the predicted flux may attain any value within these areas. Abbreviations: **(A)** ADH, alcohol dehydrogenase; **(B)** PDC, pyruvate decarboxylase; **(C)** PPS, PEP synthase; **(D)** ME, malic enzyme; **(E)** PYK, pyruvate kinase; **(F)** RuBisco, ribulose-1,5- bisphosphate carboxylase/oxygenase; **(G)** maint, basal non-growth related maintenance; **(H)** PEPC, PEP carboxylase; **(I)** CB6F, cytochrome b6f complex inc. Q-cycle; **(J)** ATPase, adenosinetriphosphatase; **(K)** ENO, enolase; **(L)** MDH, malate dehydrogenase.

Figure 5 shows selected examples of flux changes that result from the transition of a growth-only phenotype to a production-only phenotype for the synthesis of ethanol. Following the transition experiments described in Figure 3, and starting with a growth-only phenotype, the growth rate is gradually decreased. All remaining resources are directed toward the synthesis of ethanol, with maximal ethanol production as the objective of the computational optimization problem. All flux changes along the transition are recorded. Shaded areas indicate flux variability, that is, regions of possible flux values corresponding to computationally equivalent solutions. As described above, several scenarios can be distinguished. We observe that the majority of fluxes decrease, as expected in a transition from the multi-product synthesis of biomass toward the synthesis of a single product. Specifically, we observe a decreasing flux for 379 (of 698 total in the network, excluding isoenzymes, and of 505 minimally required for the synthesis of biomass) reactions, of which 371 attain a zero flux in the production-only phenotype. The latter are associated exclusively with biomass synthesis and are not required for ethanol synthesis. In contrast, a total of 105 reactions increase in flux during the transition, 63 of which already have a non-zero flux during growth. A total of 63 reaction show no appreciable change in flux, where a change is defined as at least a 5% difference in the median flux between growth and product synthesis.

Reactions that increase their flux value, starting with zero flux in a growth-only phenotype, include the synthesis pathway itself, in particular, the reactions alcohol dehydrogenase (ADH, Figure 5A) and pyruvate decarboxylase (PDC, Figure 5B). An interesting observation is the increase in Rubisco (Figure 5F). While we neglect a sink effect such that removal of products increases photosynthetic flux, the absence of biomass formation in the production phenotype is already sufficient to induce an increased carbon fixation flux, making use of those resources that are not used for biomass formation. In addition to these straightforward examples, also a number of seemingly unrelated reactions increase their predicted flux value during the transition to ethanol synthesis. These flux changes are part of ATP-wasting cycles, and reflect the fact that the linear electron chain results in a higher ATP/NADPH ratio than required for the synthesis of ethanol. As the constraint-optimization problem enforces a fully balanced metabolism, excess ATP *must* be hydrolyzed in the production-only phenotype. Examples are the increased flux through the ATP maintenance reaction (Figure 5G), the futile cycles induced by the reactions PEP synthase (PPS, Figure 5C), pyruvate kinase (PYK, Figure 5E), malic enzyme (ME, Figure 5D), and PEP carboxylase (PEPC, Figure 5H). Alternative solutions to adjust the ATP/NADPH ratio include less utilization of the Q-cycle (CB6F, Figure 5I) and less ATP synthesis (ATPase, Figure 5J). The full set of changes in reaction fluxes is provided as supplementary information. Overall, our findings are in good agreement with and confirm the analysis of Erdrich et al. (2014) who show that the ATP/NADPH ratio is a major target for possible modifications and that ATP-wasting is a suitable strategy to enforce the use of ethanol as a sink for NADPH. Most of the reactions that show increased flux during ethanol synthesis are indeed part of ATP-wasting cycles to ensure that the ATP/NADPH ratio remains compatible with the ratio provides by photosynthetic linear electron flow.

### 2.5 Metabolic transitions for the synthesis of ethylene

**Figure 6:**
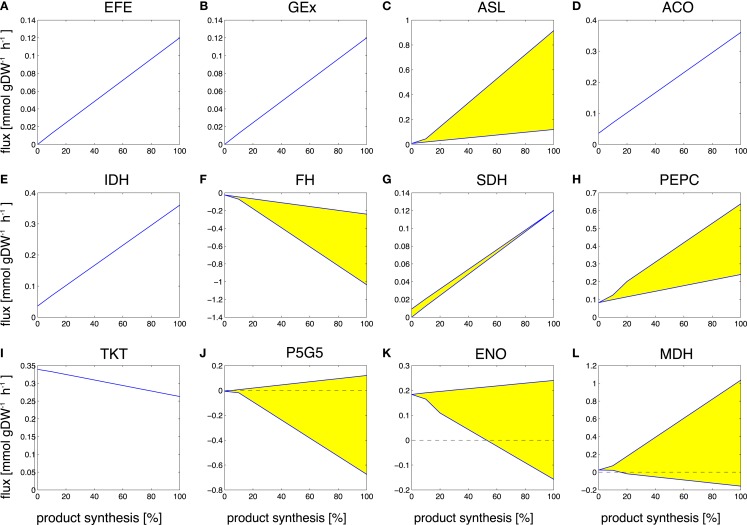
**Examples of predicted reaction fluxes during the transition from a growth-only phenotype to a production-only phenotype for ethylene**. Yellow areas correspond to non-unique solutions (flux variability). Abbreviations: **(A)** EFE, ethylene-forming enzyme; **(B)** GEx, guanidine export; **(C)** ASL, argininosuccinate lyase; **(D)** ACO, aconitase; **(E)** IDH, isocitrate dehydrogenase; **(F)** FH, fumarate hydratase; **(G)** SDH, succinate dehydrogenase; **(H)** PEPC, PEP carboxylase; **(I)** TKT, transketolase; **(J)** P5G5, conversion pyrroline-5-carboxylate + H2O ↔ glutamate 5-semialdehyde (non-enzymatic); **(K)** ENO, enolase; **(L)** MDH, malate dehydrogenase. Reactions that are predicted to increase in flux but are not part of the synthesis pathway are suggested targets for modification.

Despite its rather unfavorable stoichiometric properties, ethylene remains as a relevant compound for cyanobacterial biotechnology. Most current efforts to synthesize ethylene in cyanobacteria are based on the ethylene-forming enzyme (EFE), a rather enigmatic enzyme that synthesizes ethylene from 2-oxoglutarate (2OG) and arginine (Eckert et al., 2014). Correspondingly, the observed changes in reaction fluxes during the transition from growth to the synthesis ethylene exhibit more complex patterns than observed for ethanol. Examples of changes in selected reaction fluxes are shown in Figure 6. Again, the majority of reaction fluxes (372 of 698 total in the network, excluding isoenzymes, and of 505 minimally required for the synthesis of biomass) decrease during the transition to a production-only phenotype, reflecting the fact that the overall number of reactions required for ethylene synthesis is less than the number of reactions required for biomass synthesis. Of these 372 reactions, only 12 retain a non-zero flux in the production-only phenotype, whereas the flux of 360 reactions decreases to zero. These reactions are potential candidates for down-regulation, as their capacity in the wildtype organism is likely to exceed the capacity required for ethylene synthesis. In contrast, a total of 112 reactions increase their median flux during the transition to the production-only phenotype. Of these 112 reactions, 70 reactions already carry a non-zero median flux in the growth-only phenotype, whereas 42 increase from zero to their final value for ethylene production. The latter again include the synthesis steps itself, the ethylene-forming enzyme (EFE, Figure 6A), as well as ethylene export (GEx, Figure 6B).

Of more interest, however, are those reactions that increase in flux but are not part of the of the core synthesis pathway itself. We observe an increase in the supply of arginine via the argininosuccinate lyase (ASL, Figure 6C) and aconitase (ACO, Figure 6D), as well as an increase in the supply of 2-oxoglutarate via isocitrate dehydrogenase (IDH, Figure 6E), as well as removal of fumarate via the fumarate hydratase (FH, Figure 6F). Highly relevant are the observed changes in the succinate dehydrogenase (SDH, Figure 6G) and PEP carboxylase (PEPC, Figure 6H). These are crucial reactions to re-model the adjacent metabolism for the production of ethylene, but neither reaction is part of the immediate production pathway itself. Hence, these reactions are easily overlooked as suitable modification targets. Further, we observe changes in the transketolase (TK, Figure 6I), in the enolase (ENO, Figure 6K), and the malate dehydrogenase (MDH, Figure 6L), all associated to reduce the ATP yield of metabolism. Finally, we note changes in the reactions concerned with removal of reaction products, in particular, the conversion of pyrroline-5-carboxylate to glutamate 5-semialdehyde (P5G5, Figure 6J). The full set of changes in reaction fluxes is provided as supplementary information.

Overall, many of the dominant flux changes during the transition to the synthesis of ethylene are due to the necessity to provide the precursors arginine and 2OG, as well as to remove the by-products succinate and P5C. In particular, the increased flux through succinate dehydrogenase and through the PEP carboxylase point to promising target for further modifications. To a minor extent, we also observe a slight increase in ATP-wasting cycles, similar to the case of ethanol, but far less pronounced.

### 2.6 Metabolic transitions for the synthesis of propane

**Figure 7:**
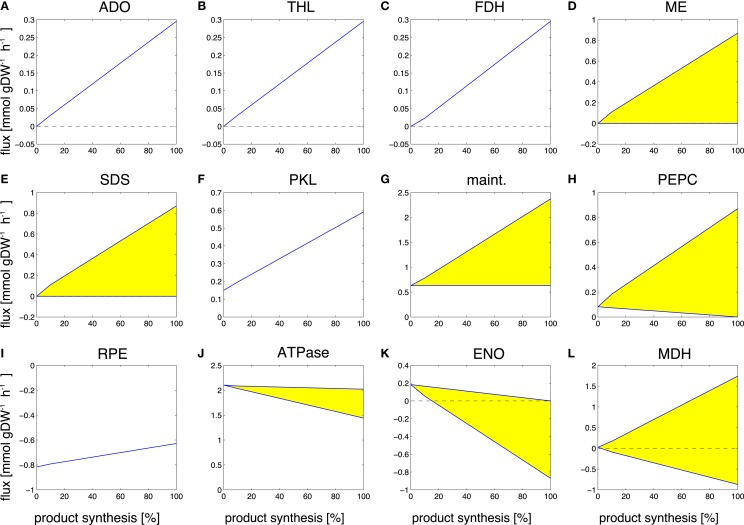
**Examples of predicted reaction fluxes during the transition from a growth-only phenotype to a production-only phenotype for propane**. Yellow areas indicate flux variability. Abbreviations: **(A)** ADO, aldehyde deformylating oxygenase; **(B)** THL, thiolase; **(C)** FDH, formate dehydrogenase; **(D)** ME, malic enzyme; **(E)** SDS, serine/threonine deaminase; **(F)** PKL, phosphoketolase; **(G)** maint, basal non-growth related maintenance **(H)** PEPC, PEP carboxylase; **(I)** RPE, ribulose-phosphate 3-epimerase; **(J)** ATPase, adenosinetriphosphatase; **(K)** ENO, enolase; **(L)** MDH, malate dehydrogenase.

Our final case study is the cyanobacterial synthesis of propane, a three-carbon alkane with manifold favorable properties and a main constituent of liquid petroleum gas (LPG). As yet, synthesis of propane by cyanobacteria is not established, but synthesis feasible in *Escherichia coli* (Kallio et al., 2014) and likely also feasible in cyanobacteria. Performing the computational transition experiment, as described above, results in a total of 388 reactions that decrease flux during the transition to propane synthesis, 370 of which cease to have flux in a production-only phenotype and only 18 have residual flux also in the production-only phenotype. A total of 92 reaction fluxes increase during the transition to propane synthesis, 45 of which already have a non-zero flux during WT growth, whereas 47 increase from zero to their final value for propane production. Figure 7 shows selected examples of differentially utilized reaction fluxes.

Reaction fluxes that increase during the transition include the synthesis steps itself, as well as the recycling of formate, a by-product of the aldehyde deformylating oxygenase (ADO) reaction. Again, of particular interest are those reactions that increase in flux but are not part of the of the core synthesis pathway. These reactions represent key targets for modification, as they constitute potential bottlenecks for the synthesis of propane. Among the predicted changes observed in the transition from growth to propane synthesis are the aldehyde deformylating oxygenase (ADO, Figure 7A) and the thiolase (THL, Figure 7B) as examples of reactions of the product pathway. The increase in the formate dehydrogenase (FDH, Figure 7C) is due to the necessity to recycle the product formate into metabolism. Examples of other reactions that exhibit a changes flux are the malic enzyme (ME, Figure 7D), the serine/threonine deaminase (SDS, Figure 7E), the phosphoketolase (PKL, Figure 7F), the unspecific hydrolyzation of ATP (maintenance, Figure 7G), PEP carboxylase (PEPC, Figure 7H), the ribulose-phosphate 3-epimerase (Figure 7I), a decreasing ATPase (ATPase, Figure 7J), and enolase (ENO, Figure 7K), as well as a broader range for the malate dehydrogenase (MDH, Figure 7L). The observed transitions reveal a complex pattern that is not straightforward to interpret. Most reactions with increased flux are again associated with ATP-wasting cycles. As in the case of ethanol synthesis, the reason why these reactions carry higher flux in the production phenotype is due to the predicted ATP/NADPH ratio: for the synthesis of propane, this ratio is significantly below that of either biomass synthesis or the linear electron transport chain (0.92 versus 2.13 versus 1.3, respectively). Hence, the observed flux changes in the transition experiment serve to remove excess ATP from metabolism, either by overexpressing ATP-wasting cycles or by utilizing less ATP-efficient pathways (for example, by a decrease in ATPase, Figure 7J). Similar as for the case of ethanol synthesis, the ATP/NADPH can be considered a prime target for enhancing propane synthesis.

## 3 Discussion

Computational network reconstructions and metabolic modeling offers significant potential to identify and guide suitable strain design strategies for cyanobacterial biotechnology. In particular, recent genome-scale metabolic reconstructions of cyanobacterial metabolism provide an increasingly comprehensive view on the cyanobacterial metabolic network. These reconstructions are therefore a valuable knowledge base and allow for a systematic computational analysis of key stoichiometric properties of cyanobacterial biofuel production.

In this work, we investigated two aspects of cyanobacterial product synthesis. First, we summarized several basic stoichiometric properties related to the synthesis of various cyanobacterial products for which the biological feasibility of synthesis by cyanobacteria has already been demonstrated or is likely to be feasible. Second, we performed *in silico* transition experiments to mimic the transition from a growth-only phenotype to an envisioned production phenotype. The *in silico* transition experiments highlight necessary adaptations in metabolic fluxes and therefore point to potential bottlenecks in product synthesis. In particular, the synthesis of a desired product must be compatible with the properties of the host metabolism. For low overall synthesis fluxes, as typically encountered in current pilot studies of cyanobacterial biotechnology, such compatibility is usually no major issue and only requires minor adaptations in growth-related metabolism. A host metabolism whose flux distribution and reaction usage is dominated by synthesis flux toward a desired product, however, has to accommodate significantly larger perturbations of its native flux pattern.

In this respect, a first issue is the compatibility of the ATP/NADPH ratio required for product synthesis, as compared to the ratio required for cellular growth. For most currently considered small molecule products, the ATP/NADPH ratio is below the value estimated for cellular growth, hence excess ATP must be hydrolyzed or channeled into other cellular processes. Since the minimal ATP/NADPH ratio is limited by the linear electron transport chain, alternative sinks for ATP are necessary and typically decrease the total productivity of the organisms. Another relevant issue is the provision of co-factors and the reintegration of by-products of product synthesis. As shown, complex reaction stoichiometries, as encountered for the synthesis of ethylene, induce significant changes in the fluxes of adjacent pathways, such as the TCA cycle in the case of ethylene. As the reactions required for reintegration of by-products and provision of co-factors are typically not part of the synthesis pathways themselves, the necessity to increase the capacity of such reactions might be easily overlooked. In some cases, it might even be a beneficial strategy to introduce further synthetic reactions that improve recycling of by-products, such as formate (Bar-Even et al., 2013), and thereby prevent or reduce carbon loss.

In general, *in silico* transition experiments, as presented here, allow for a comparatively quick and inexpensive view on required flux changes for a desired production phenotype. We note that the *in silico* flux distribution should not be understood as predictions that arise from heterologous expression of product-forming pathways. Rather the computationally obtain flux solutions must be interpreted as desirable target states that may or may not be attained using further targeted modifications of metabolism. It then remains the prime task of metabolic engineering to push cellular metabolism toward the desired phenotype, while retaining viability of the cell and high metabolic productivity. Notwithstanding the fact that computational modeling cannot foresee the manifold obstacles encountered in this endeavor, we are confident that such computational analyses will play increasing roles in a future green biotechnology. Specifically, large-scale metabolic reconstructions are rapidly increasing in scope and quality, and provide an unbiased view on stoichiometric necessities that occur during the transitions from wildtype growth to a production phenotype. In this sense, computational modeling can provide a useful input to guide metabolic modifications and thereby augment current experimental efforts in cyanobacterial biotechnology.

## 4 Materials and Methods

### 4.1 The metabolic network of *Synechocystis* sp. PCC 6803

All simulations are based on a previously published genome-scale stoichiometric network model of the cyanobacterium *Synechocystis* sp. PCC 6803 (Knoop et al., 2013; 2010). The current reconstruction encompasses 780 metabolic reactions (814 including reaction specific for fuel synthesis and export) and 601 metabolic species. The network was augmented with synthesis reactions for twelve different products: ethanol, ethylene, lactate, propane, 1-butanol, isoprene, 1-octadecanol, heptadecane, pentadecane, butane-2,3-diol, isobutyraldehyde, and isobutanol. See Table 3 for an overview of molecular composition and lower heating value (LHV). The specific synthesis pathways, which are not part of WT metabolism, have been sourced from current literature and are summarized in Table 4. In addition to synthesis reactions also export reactions for each of the analyzed bioproducts are added to the network. For ethanol, synthesis was enforced via the pyruvate decarboxylase (PDC) and aldehyde dehydrogenase (ADH), as described in Deng and Coleman (1999). A straightforward computational optimization would otherwise choose a flux solution via acetate. The resulting metabolic network model, including all augmented reactions, is provided as a supplemental data.

**Table 3:** **Elemental stoichiometry and lower heating value (LHV) for selected cyanobacterial bioproducts**.

Bioproduct		LHV [kJ mol^−1^]
Ethanol	C_2_H_6_O	1370
Ethylene	C_2_H_4_	1410
Lactate	C_3_H_6_O3	1362
Propane	C_3_H_8_	2220
1-Butanol	C_4_H_10_O	2670
Isoprene	C_5_H_8_	3190
1-Octadecanol	C_18_H_38_O	11820
Heptadecane	C_17_H3_6_	11350
Pentadecane	C_15_H_32_	10050
Butane-2,3-diol	C_4_H_10_O_2_	2460
Isobutyraldehyde	C_4_H_8_O	2470
Isobutanol	C_4_H_10_O	2670

**Table 4:** **Synthesis pathways for cyanobacterial bioproducts**.

	Enzyme	ID	Reaction	Reference
**Ethanol**				
Pyruvate decarboxylase	PDC	Bi0001	Pyruvate ⇒ Acetaldehyde + CO_2_	Deng and Coleman (1999)
**Ethylene**				
Ethylene-forming enzyme	EFE	Bi0005	3 2-OG + 3O_2_ + l-Arginine ⇒ 2 Ethylene + 7CO_2_ + Succinate + Guanidine + (S)-1-P5C + 3H_2_O	Takahama et al. (2003)
**Propane/1-butanol**				
Crotonase	CRT	Bi0006	(R)-3-Hydroxybutanoyl-CoA ⇒ Crotonoyl-CoA + H_2_O	Lan and Liao (2011); Lan et al. (2013)
Transenoyl-CoA reductase	TER	Bi0007	Crotonoyl-CoA + NADH + H^+^ ⇒ Butanoyl-CoA + NAD^+^	Lan and Liao (2011); Lan et al., 2013)
CoA-acetylating propionaldehyde dehydrogenase	PDUP	Bi0008	Butanoyl-CoA + NADH + H^+^ ⇒ Butyraldehyde + CoA + NAD^+^	Lan and Liao (2011); Lan et al. (2013)
**Propane**				
Aldehyde deformylating oxygenase	ADO	Bi0009	Butyraldehyde + 4 H^+^ + O_2_ + 4 ferredoxin_red_ ⇒ Propane + Formate + H_2_O + 4ferredoxin_OX_	Khara et al. (2013)
**1-butanol**				
Alcohol dehydrogenase	ADH	Bi0010	Butyraldehyde + NADPH + H^+^ ⇒ 1-Butanol + NADP^+^	Lan and Liao (2011); Lan et al. (2013)
**Isoprene**				
Isoprene synthase	ISPS	Bi0011	Dimethylallyl diphosphate ⇒ Isoprene + Diphosphate	Lindberg et al. (2010)
**1-Octadecanol/heptadecane**				
Acyl-ACP reductase	AAR	Bi0012	Octadecanoyl-[acp] + NADPH + H^+^ ⇒ Octadecanal + ACP + NADP^+^	Schirmer et al. (2010)
**1-Octadecanol**				
Alcohol dehydrogenase	ADH	Bi0014	Octadecanal + NADPH + H^+^ ⇒ 1-Octadecanol + NADP^+^	Vidal et al. (2009)
**Heptadecane**				
Aldehyde deformylating oxygenase	ADO	Bi0016	Octadecanal + 4 H^+^ + O_2_ + 4 ferredoxin_red_ ⇒ Heptadecane + Formate + H_2_O + 4 ferredoxin_OX_	Khara et al. (2013)
**Pentadecane**				
Acyl-ACP reductase	AAR	Bi0013	Hexadecanoyl-[acp] + NADPH + H^+^ ⇒ Hexadecanal + ACP + NADP^+^	Schirmer et al. (2010)
Aldehyde deformylating oxygenase	ADO	Bi0017	Hexadecanal + 4 H^+^ + O_2_ + 4 ferredoxin_red_ ⇒ Pentadecane + Formate + H_2_O + 4 ferredoxin_OX_	Khara et al. (2013)
**2,3-Butanediol**				
2-Acetolactate decarboxylase	ALDC	Bi0018	2-Acetolactate ⇒ (R)-Acetoin + CO_2_	Oliver et al. (2013)
Alcohol dehydrogenase	ADH	Bi0019	(R)-Acetoin + NADH + H^+^ ⇒ (R,R)-Butane-2,3-diol + NAD^+^	Oliver et al. (2013)
**Isobutyraldehyde**				
2-Ketoacid decarboxylase	KDC	Bi0002	3-Methyl-2-oxobutanoic acid ⇒ Isobutyraldehyde + CO_2_	Atsumi et al. (2009)
**Isobutanol**				
Alcohol dehydrogenase	ADH	Bi0003	Isobutyraldehyde + NADH + H^+^ ⇒ Isobutanol + NAD^+^	Atsumi et al. (2009)

*The respective enzymatic steps are introduced into the stoichiometric reconstruction of Synechocystis *sp*. PCC 6803. The reaction ID denotes the identifier of the reaction within the reconstructed network*.

*2OG, 2-oxoglutarate; (S)-1-P5C, (S)-1-pyrroline-5-carboxylate*.

### 4.2 Synthesis of ethylene

The synthesis of ethylene via the ethylene-forming-enzyme (EFE) yields guanidine as a co-product. The fate of guanidine is unknown and has not been discussed in previous works (Eckert et al., 2014). No straightforward enzymatic reactions exist to reintegrate guanidine into cyanobacterial metabolism. For all calculations described in the main text, therefore, an export transport reaction for guanidine was added to the model. Alternatively, guanidine (CH5N3) might non-enzymatically decompose into urea (CH4N2O) and ammonia (NH3), with timescales comparable to product formation (Lewis and Wolfenden, 2014). Full reintegration of guanidine into metabolism result in slightly modified values compared to those reported in Table 1. In this case, only 44 photons are used per molecule of ethylene, whose synthesis then requires 18 molecules of ATP and 13 molecules of NADPH. The maximal synthesis flux changes to 0.354 mmol gDW^−1^h^−1^, resulting in a maximal yield of 0.449 J gDW^−1^h^−1^ for a constant light input of 15.57 μmol photons gDW^−1^h^−1^. The modified yield remains significantly below the values obtained for all other products. All conclusions given in the main text remain valid and apply to both scenarios.

### 4.3 Flux-balance analysis

All flux distributions, including the estimation of maximum yield and corresponding stoichiometric properties have been calculated using the toolbox COBRA (v2.05) (Schellenberger et al., 2011), running in the matlab environment (The MathWorks, Inc., Natick, MA, USA). Flux-balance analysis is based on knowledge of reaction stoichiometries only. Given a limited input flux, FBA seeks to computationally estimate the maximal possible output flux, usually the synthesis of biomass as specified by a biomass objective function (BOF) that defines the cellular constituents in their correct proportion. We note that FBA is only concerned with stoichiometric yield. Nonetheless, the results are often reported in units of a (growth) rate that is obtained by multiplying the estimated maximal yield with the given input flux rate. The advantages of FBA reside (i) in the fact that the estimation of stoichiometric yield is not computationally expensive and feasible even for large models involving hundreds of reactions; as well as (ii) the fact that only stoichiometric information and no kinetic parameters are required. The latter are usually not available even for small reaction networks. In contrast, stoichiometric information is readily and reliably available for a substantial proportion of annotated enzymes (Steuer et al., 2012). We emphasize that FBA is subject to inherent limitations and issues like product inhibition, potential toxicity of by-products, the impact of localization and compartmentalization, as well as impact of molecular crowding are outside the scope of the modeling framework.

### 4.4 Parameters and computational details

The following constraints were applied to the model of *Synechocystis* sp. PCC 6803: light input serves as sole external energy source, and ${\mathrm{HCO}}_{3}^{-}$, respectively, CO_2_, as sole carbon source (phototrophic growth). ${\mathrm{HCO}}_{3}^{-}$ can be taken up by either by ATP- or Na^+^-dependent transport mechanisms. The amount of available light flux was chosen such that the model supports a maximum growth rate of 0.0289 h^−1^, corresponding to a doubling time of approximately 24 h. For the calculation of stoichiometric properties and trade-off analysis, Rubisco carboxylase and oxygenase (PP0011) were decoupled, resulting in two separate reactions (MO0007 and MO0008). No energy consuming side reactions, such as maintenance, evolution of reactive oxygen species, respiration, or the Mehler-like reaction, were considered for the estimation of maximal stoichiometric product yield, as well as biomass yield, shown in Table 1. Table 1 further shows the stoichiometric amount of photons, oxygen, NADPH, ATP, and carbon molecules needed to synthesize 1 mmol of the respective product, as obtained from the computational optimization problem. Therein, ATP requirements are estimated from the flux through the ATPase reaction. NADPH requirements are obtained from flux through FNR reaction minus flux through NDH-1. It is emphasized that the precise ATP/NADPH ratio for biomass synthesis is unknown. The estimate given in Table 1 depends on several assumptions with respect to ATP expenditure of carbon import and the exact representation of cyclic electron transport in the model, and should not be interpreted as a precise estimate. Rather, it serves as a reference value for product synthesis, whose estimated ATP/NADPH ratio subject to similar uncertainties. In particular, the estimated value for biomass synthesis is consistently larger than the ratio estimated for most product syntheses, independent of computational details. Photons in Table 1 indicate total photons and are distributed between the two photosystems as the simulation demands. When assuming photons of wavelength 680 nm only, the total photon influx in Table 1 (last column) corresponds to an energy uptake of approximately 2.7 J gDW^−1^h^−1^. The lower heating values (LHVs) at room temperature have been extracted from the NIST-webbook of chemistry (Linstrom and Mallard, 2014), with the exception of the value for lactate. For the estimation of the trade-off from biomass to product synthesis, the minimally required growth rate was set stepwise to values between 0 and 100% of the maximum achievable rate. Given a constant light input and the required growth rate, the rate of product synthesis was maximized, thereby directing all resources beyond the enforced growth rate toward product synthesis.

### 4.5 Flux variability and transition experiments

For the analysis of the transition from growth to product synthesis, additional constraints were taken into account to reflect the initial WT state of metabolism. We note that these additional constraints increase the number of active reactions required for biomass synthesis, as compared to the value reported in Table 1. The Rubisco-reactions carboxylase and oxygenase (PP0011) were now coupled, forcing 3% of flux through the oxygenase. A basal respiration (PR0010, cytochrome *c* oxidase) using 10% of the oxygen evolution of photosystem II (PR0043) was assumed to take place. Likewise, the rate of the Mehler-like reaction (PR0033) was fixed to 10% of the initial oxygen evolution of photosystem II (PR0043). The evolution of reactive oxygen species (ROS) from the Mehler-reaction (PR0032) and from PS II (PR0034) were both set to 0.5% of the electrons converted in the respective photosystem (PR0002, PR0003). The basal ATP-demand for general cellular maintenance (GE0001) was set to 0.6312 mmol gDW^−1^h^−1^. Isoreactions have been excluded in the analysis of flux variability. For the transition experiment, a constant light availability of 18.0 mmol photons gDW^−1^h^−1^ was assumed, again corresponding to a maximal doubling time of approximately 24 h when considering the additional constraints. For detailed discussion of metabolic constraints used for flux estimation see Knoop et al. (2013).

Simulating the transition from growth to product synthesis was performed identical to trade-off analysis. Our overall strategy is similar to the approach developed by Choi et al. (2010), therein applied to identify gene amplification targets for the improvement of lycopene production in *E. coli*. This and similar analyses have as yet not been applied to phototrophic product synthesis. We go beyond a single product and focus on a comparison between different target products. To estimate the transition from a WT phenotype to a production-only phenotype, the enforced growth rate was stepwise decreased from 100 to 0% of the maximum rate. Subsequently, product synthesis was maximized using a linear programing framework. For each simulation step, the variability of every metabolic rate was determined. For further investigation, we focus on reactions where the mean of the estimated flux range, is either larger than 10^−6^ mmol gDW^−1^h^−1^ for 100% biomass or larger than 10^−4^ mmol gDW^−1^h^−1^ for 100% product synthesis. Reactions for which the overall change of the mean flux rate from 100% biomass synthesis to 100% product synthesis was <5% were marked as no change. Reactions with an increment of the flux rate of larger than 5% were either marked as “flux increase” (either “from flux” or “from zero,” where the former indicates a non-zero flux already in the growth phenotype). Reactions with an decreasing flux rate were assigned as “flux decrease,” either “to flux” and “to zero,” depending on whether the mean of the flux rate at 100% product synthesis was larger than 10^−4^ mmol gDW^−1^h^−1^. The label “sign change” indicates that the product of the minimal flux rate for 100% biomass formation and the minimal flux rate for 100% product synthesis, or the maximal flux rate for 100% biomass formation and the maximal flux rate for 100% product synthesis is negative. We emphasize that the predicted flux solutions optimized for high product synthesis are not predictions of actual flux solutions after heterologous expression of product-forming pathways. Rather they must be interpreted as desirable target states that may or may not be attained using further targeted modifications of metabolism. It has been shown that that in practice high carbon partitioning toward product synthesis may also have detrimental effects that are not accounted for by flux-balance analysis (Oliver and Atsumi, 2015).

## Author Contributions

RS devised the research and prepared Figures 2 and 4. HK carried out all simulations and prepared all remaining figures. HK and RS wrote the main manuscript text. All authors reviewed and approved the manuscript.

## Conflict of Interest Statement

The authors declare that the research was conducted in the absence of any commercial or financial relationships that could be construed as a potential conflict of interest.

## Supplementary Material

The Supplementary Material for this article can be found online at http://journal.frontiersin.org/article/10.3389/fbioe.2015.00047

- **File Metabolic_Network_Supplementary_File1.xml:** the network file used in the analysis (SBML).
- **File Results_Transition_Analysis_Supplementary_File2. xls:** full results of the transition analysis (xls).
- **File Knoop2015_Simulation_Constraints_Supplementary_File3.xls:** additional constraints used in the computational simulations (xls).
